# *Ex vivo* perfusion model of mouse liver and its application to analyze the effects of OCT1 deficiency

**DOI:** 10.3389/fphar.2025.1629421

**Published:** 2025-11-14

**Authors:** Vincent Rönnpagel, Felix Morof, Giuliano Ciarimboli, Markus Grube, Marleen J. Meyer-Tönnies, Mladen V. Tzvetkov

**Affiliations:** 1 Department of General Pharmacology, Department of General Pharmacology, Institute of Pharmacology, Center of Drug Absorption and Transport (C_DAT), University Medicine Greifswald, Greifswald, Germany; 2 Experimental Nephrology, Medicine Clinic D, Münster University Hospital, Münster, Germany

**Keywords:** mouse liver perfusion, OCT1, drug transporter, pharmacokinetics, first-pass simulation, codeine, morphine, proguanil

## Abstract

**Introduction:**

The liver plays a critical role in drug pharmacokinetics. In *in vivo* experiments, it is difficult to isolate the liver’s contribution to drug systemic concentrations from that of the intestine and kidneys. Rat liver perfusion is well-established for studying liver-specific effects. However, rats are not easily genetically manipulated, complicating analyses of individual drug transporters and metabolizing enzymes. This study aimed to establish an *ex vivo* liver perfusion model in mice and to apply it to analyze the effects of mOct1 on drug metabolism.

**Methods:**

After euthanizing, the liver of 6- to 28-week-old mice was perfused via an indwelling venous catheter in the portal vein as entry and into the caudal vena cava toward the heart as exit. Perfusion solutions were prewarmed to 42 °C and pumped at 2 mL/min. First, HBSS supplemented with 0.5 mM EDTA was used to exsanguinate the liver, followed by HBSS alone and then HBSS containing the drug of interest. Drug and metabolite concentrations in the perfusates were measured by LC-MS/MS.

**Results and conclusion:**

The method enables reproducible and reliable perfusion of mouse livers. We applied it to study the effects of Oct1 knockout on drug metabolism. Oct1 knockout affected the first-pass metabolism of codeine, including the formation of the metabolites morphine and morphine-3-glucuronide, as well as the first-pass metabolism of proguanil and the formation of cycloguanil. The model is applicable to any mouse strain, genetic background, and substrate of interest and is thus applicable to a wide variety of research questions.

## 1 Introduction

The liver plays a crucial role in drug metabolism and, along with the gut and kidneys, significantly influences a drug’s pharmacokinetics. The liver affects pharmacokinetics both during the first-pass metabolism and during regular turnover in the systemic circulation. During first-pass metabolism, isolating the liver’s contribution from intestinal resorption and metabolism *in vivo* is very difficult. In the systemic turnover of blood, distinguishing the liver’s contribution to drug metabolism and excretion from that of the kidneys and other organs is also difficult.

Primary human hepatocytes are a commonly used model system for *in vitro* analyses of hepatic metabolism (Sun et al., 2019; Bonanini et al., 2024; Hewitt et al., 2007; Vilas-Boas et al., 2019; Yang et al., 2016). However, they are laborious to isolate and their availability depends on human organ donations. Two additional limitations include, on the one hand, the rapid downregulation of metabolizing enzymes and transporters once cultured (Tchaparian et al., 2011; Sidler Pfändler et al., 2004). On the other hand, up to 40% of the cells in the liver are not hepatocytes, and their functions are not accounted for in this model (Kmieć, 2001; Vekemans and Braet, 2005; Clark et al., 2014). The effects of multi-dimensional structures, such as the space of Disse or the oxygen gradient from the portal triad to the central vein (Kietzmann, 2017), could also not be modeled by using primary hepatocytes only.

Established human cell lines of hepatic origin, like HepG2 and HuH7, address the challenges associated with primary material. However, these tumor-derived cell lines exhibit reduced or completely lack expression of key drug transporters and metabolizing enzymes (Wilkening et al., 2003; Olsavsky et al., 2007; Gerets et al., 2012; Jouan et al., 2016; Malinen et al., 2019). Cell lines derived from immortalized human hepatocytes, like HepaRG (Aninat et al., 2006; Guillouzo et al., 2007), especially in combination with organoid approaches (Bell et al., 2017), offer promising alternatives, yet they still fail to fully represent drug transport, metabolism, and their regulation (Tascher et al., 2019).

The rat liver perfusion model is well-developed and is widely used for analyzing drug pharmacokinetics (Heylen et al., 2025) and physiological processes in the liver (Ross et al., 1967; Gores et al., 1986). However, rats are not so commonly and easily genetically manipulated. Therefore, a mouse liver perfusion model may be more useful for directly addressing the effects of key drug transporters and metabolizing enzymes. As early as 1996, a humanized mouse model was used to reveal the role of CYP2E1 in the toxification of paracetamol (Lee et al., 1996). Since then, numerous genetically engineered models—including knockout and humanized models of CYP enzymes, regulatory factors such as PXR and CAR, as well as efflux transporters—have been developed and applied to investigate drug pharmacokinetics, toxicity, and drug–drug interactions (Hannon and Ding, 2022). More recently, advanced models such as the 8HUM mouse, which expresses up to 32 humanized CYP enzymes, have become available (Kapelyukh et al., 2025).

This implicates a downscaling that may need modifications of the well-established rat liver perfusion model. There are various models that can be used as a guide for this. For example, the isolation methods for hepatocytes and non-parenchymal cells (Seglen, 1976; Charni-Natan and Goldstein, 2020) or other short time perfusion models (Salous et al., 2013; Lee et al., 2009).

Organic cation transporter 1 (OCT1, *SLC22A1*) can transport multiple drugs into the liver and thus play a limiting role for their plasma concentrations (Matthaei et al., 2016; 2019; Tzvetkov et al., 2011; 2013; 2018; Stamer et al., 2016). OCT1 is highly expressed in the sinusoidal membrane of hepatocytes. In humans, this is the site with the strongest expression of this transporter (Nies et al., 2009; Zhang et al., 2024). In rodents, including mice, Oct1 is strongly expressed both in hepatocytes and basolaterally in the proximal tubules (Karbach et al., 2000; Jonker and Schinkel, 2004). These expression differences make it difficult to model OCT1 effects in humans based on Oct1 knockout mice alone. The group of Alfred Schinkel developed the single Oct1 and the double Oct1/Oct2 knockout mice (Jonker et al., 2001; 2003). Currently, only the double knockout is commercially available (Taconic Biosciences) and widely used in mouse studies (Sprowl et al., 2014; Wang et al., 2002; Morse et al., 2021).

In experiments with oral drug administration in living animals, it is difficult to separate the first-pass effects of the liver from those of the intestine. Both organs can influence drug transport and metabolism (Drozdzik et al., 2020; Zamek-Gliszczynski et al., 2022). OCT1, in particular, is expressed not only in the liver but also in mice intestine and kidney (Wenzel et al., 2021; Jonker et al., 2003). Moreover, the commercially available Oct1 knockout mice are actually double knockouts (Oct1 and Oct2, taconic biosciences–model #6622), which significantly affects renal drug clearance for OCT substrates. This makes it challenging to interpret data from drug administration in live animals when the aim is to analyze Oct1 only. The model described here allows the use of these commercially available animals to better assess liver-specific effects typical of human OCT1.

Here, we describe a method to perfuse the mouse liver *ex vivo* and demonstrate its application to simulate the effects of mouse Oct1 on the plasma concentrations of drugs. We show the impact of Oct1 knockout on the first-pass metabolism of codeine, including the formation of its metabolites' morphine and morphine-3-glucuronide, as well as on the first-pass metabolism of proguanil and the formation of its metabolite cycloguanil. The drugs were selected as known OCT1 substrates that have been shown to be affected by OCT1 polymorphisms in humans (Fukuda et al., 2013; Tzvetkov et al., 2013; Venkatasubramanian et al., 2014; Hahn et al., 2019; Matthaei et al., 2019).

## 2 Materials and equipment

### 2.1 Animals

We used male FVB mice with wild-type and Oct1/2 knockout (Oct1/2 KO) genotypes. This mouse model has been described and characterized in detail previously (Jonker et al., 2001; 2003). We confirmed both the deletion at the DNA level and the effect on Oct1 mRNA expression in our hands (Supplementary Figure S1). Please note that Oct1 mRNA levels were strongly reduced but not completely absent in the knockout mice. This is consistent with the findings of Jonker et al., who also reported residual amounts of truncated transcript that do not result in protein synthesis. The mice were aged 6–28 weeks (weight between 23.6 and 37.8 g). It is possible to use any other strain and genetic background. We successfully used the method also to analyze the C57BL/6 strain without any significant changes in performance (data not shown).

Animal experiments were conducted in accordance with European, German, and local legal requirements. At the time of liver perfusion, the animals were already deceased. All animals used were reported to the relevant authorities.

### 2.2 Reagents

The reagents used for this method are shown in Table 1.

**TABLE 1:** Reagents used in the perfusion system.

Reagent	Supplier	Catalog number
Hanks’ Balanced Salts	Merck	H4891-10X1L
Ampuwa water	Fresenius Kabi	B230673
HEPES	Carl Roth	HN77.4
Sodium hydroxide	Carl Roth	K021.1
Hydrochloric acid	Carl Roth	K025.1
EDTA	Carl Roth	8040.2
Carbogen gas	Air Liquide	P3750
Ketamine	Selectavet Dr. Otto Fischer GmbH	
Xylazine	Selectavet Dr. Otto Fischer GmbH	
70% ethanol	Carl Roth	T913.3
Lucifer Yellow CH dilithium salt	Merck	L0259
Morphine hydrochloride trihydrate	CAELO	4888-1g
Morphine-3-glucuronide	Sigma-Aldrich	M-031-1ML
Morphine-6-glucuronide	Sigma-Aldrich	M-120-1ML
Codeine-6-glucuronide	Lipomed AG	C-60-TF
Morphine-d3	Sigma-Aldrich	M-003-1ML
Codeine monohydrate	Lipomed AG	C-69-FB
Proguanil hydrochloride	Sigma-Aldrich	G7048-10mg
Cycloguanil hydrochloride	Santa Cruz Biotechnology	sc-207470
Proguanil-d6 hydrochloride	Toronto Research Chemicals	C329503
Liquid nitrogen	Air Liquide	I4195RG
Acetonitrile in LC-MS/MS grade	Avantor	9821.2500GL
Methanol in LC-MS/MS grade	Carl Roth	7342.1
Formic acid in LC-MS/MS grade	Merck	1002641000

### 2.3 General tools

The instruments used for this method are shown in Table 2.

**TABLE 2:** Instruments used in the perfusion system.

Instrument	Instrument manufacturer description	Manufacturer	Model/catalog number
Sterile filter	Minisart^®^ PES15, 0.2 µm	Sartorius	1776D ACK
pH meter	SevenEasy pH meter	Mettler-Toledo	S20
Water bath	Incubation/Inactivation Bath	GFL	1002
Pump tubing	Pump tubing	Masterflex^®^ Ismatec^®^	MFLX97616-24
Peristaltic pump	Peristaltic Pump	Ismatec^®^	ISM831A
Scale	Scale	PCE group	PCE-BDM1.5
Heating plate	Heating plate	Minitube	HT007
Syringe	Omnican®-F	B Braun	9161502S
Fixation plaster	Fixation Plaster	BSN medical	76820-00
Forceps	Curved pointed forceps	Carl Roth	2858.1
Surgical suture material	surgical suture material	Catgut	18104810
Suture material	suture material	SMI	193069
Indwelling venous catheter	26G venous catheter	Greiner Bio-One	NW261901
Stereo microscope	Mantis Compact	Vision engineering	VE4-200002
Dissecting scissor	dissecting scissor	Carl Roth	TE35.1
Centrifuge	MicroCL 17R Microcentrifuge	Thermo Fisher Scientific	75002455

### 2.4 Reagent setup

#### 2.4.1 Base medium for all solutions

First, we prepared a 10× stock solution of Hanks’ Balanced Salt Solution (HBSS). To do so, we dissolved Hanks’ salts in 80 mL of sterile water and added 10 mL of 1 M HEPES. We adjusted the pH to 7.4, filled up the solution to 100 mL with sterile water, and then sterile-filtered the solution. This stock solution was stored at 4 °C for up to 2 months.

All subsequent solutions were freshly prepared and used on the same day.

##### 2.4.1.1 Solution 1: liver exsanguination solution

The liver exsanguination solution is used prior to perfusion and is essential for achieving uniform perfusion throughout the liver. It contains EDTA to prevent blood clotting. To prepare it, 30 mL of mouse liver regeneration solution (Solution 2) was supplemented with 240 µL of EDTA (stock concentration: 0.5 M; final concentration: 4 µM). The pH was adjusted to 7.4.

##### 2.4.1.2 Solution 2: liver regeneration solution

The liver regeneration solution is used to replenish the liver with calcium after exsanguination with the chelator EDTA by using solution 1. To this end, 2 mL base medium was filled up to 15 mL with sterile water. Then, the pH was measured and adjusted to 7.4 if necessary and after that, the volume was filled up to 20 mL with sterile water.

##### 2.4.1.3 Solution 3: substrate perfusion solution

The substrate perfusion solution is the main solution used during the experiment. It was prepared by adding the drug of interest to 70 mL of liver regeneration solution (solution 2). The pH was adjusted to 7.4. Stock solutions of the substrates had been previously prepared in water and stored at −20 °C. Final substrate concentrations in the perfusion solution were 0.1 µM or 25 µM for morphine (from stock solutions of 0.1 mM or 20 mM, respectively), 25 µM for codeine (stock 20 mM), 2.5 µM or 25 µM for proguanil (stock 10 mM), and 0.3 µM for cycloguanil (stock 10 mM). All substrate perfusion solutions were treated with carbogen gas for 30 min before use.

##### 2.4.1.4 Solution 4: post-perfusion sample processing solution

The post-perfusion sample processing solution is used to prepare the samples after perfusion for LC-MS/MS analyses. It consisted of 80% acetonitrile and 20% water, supplemented with internal standards for LC-MS/MS analysis. For each perfusion experiment, 40 mL of processing solution was prepared. The internal standards and their concentrations used in this study are listed in Table 3.

**TABLE 3:** Parameters for the quantitative LC-MS/MS detection of the tested substrates.

Analyte	MRM (m/z)	CE [volt]	DP [volt]	RT [min]	Internal standard	IS MRM (m/z)	CE [volt]	DP [volt]	RT [min]	Stock [µg/mL]	Final conc. [ng/mL]
Morphine	286.2/201.1 286.2/165.1	36 54	110	4.10	Morphine-d3	289.1/201.0 289.1/165.0 289.1/181.0	36 55 49	80	4.08	10	10
Morphine-3-glucuronide	462.0/286.2	42	100	3.52							
Morphine-6-glucuronide	462.0/286.2	42	100	4.59							
Codeine	300.2/215.1 300.2/165.1	35 57	110	8.14							
Codeine-6-glucuronide	476.1/300.0 476.1/215.1	43 55	90	9.85							
Proguanil	254.1/170.1 254.1/153.1	25 39	25 39	3.50	Proguanil-d6	260.1/170.1 260.1/153.1	28 43	28 43	3.44	10	5
Cycloguanil	252.1/195.1 252.1/153.2	24 43	24 43	2.13							

MRM, multiple reaction monitoring; CE, collision energy; DP, declustering potential; RT, retention time; IS, internal standard.

## 3 Methods

### 3.1 Preparation of the perfusion system (Step I)

**FIGURE 1:**
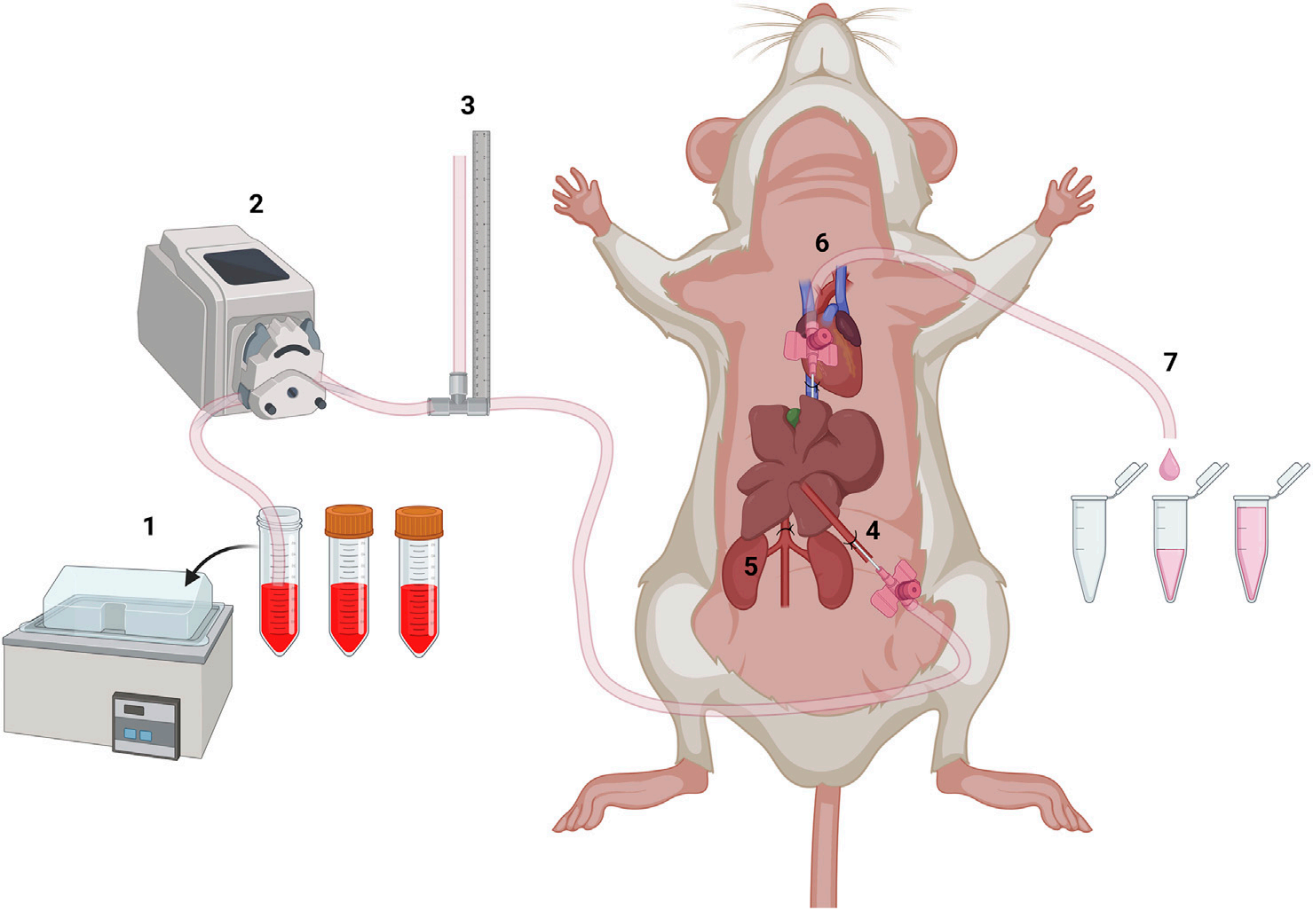
Schematic setup of the mouse liver perfusion Shown are (1) the pre-warmed solutions (42 °C), (2) the peristaltic pump, (3) the pressure monitoring, (4) the cannulated portal vein, (5) the tied caudal vena cava, (6) the cannulated caudal vena cava, and (7) the sample collection (Created with BioRender.com).

The general set-up of the system is shown in Figure 1.

1. Preheat solutions 1–3 to 42 °C in a water bath (Figure 1, position 1).
2. Insert the first pump tubing into solution 1 and connect it to the peristaltic pump.
3. Calibrate the pump to 2 mL/min (Figure 1, position 2).
4. Connect the pump tubing to a T-shaped glass tubing connector (Figure 1, position 3).
5. Fix the T-shaped glass tubing connector to the table with a fixation plaster so that one outlet runs vertically to the table.
6. Attach a second pump tubing to the vertical outlet and secure to the water bath using fixation plasters.
7. Attach a third pump tubing to the last outlet and place it back into solution 1.
8. Switch on the pump to preheat the system.
9. Preheat the heating plate to 37 °C.
10. Collect 1 mL from every solution used for later concentration measurements.

### 3.2 Preparation of the mouse and perfusion (Step II)

**FIGURE 2:**
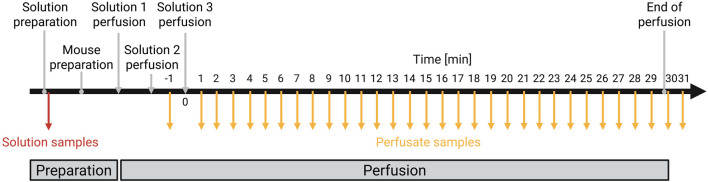
Timeline for solution administration and sample collection during liver perfusion Prepared solution samples are shown in red, perfusate samples in orange, and all steps related to the perfusion are shown in gray (Created with BioRender.com).

The sampling procedure is visualized in simplified form in Figure 2.

1. Take the mouse out of the cage and weigh it.
2. Calculate the dose for anesthesia based on the body weight: 100 μg/g ketamine and 25 μg/g xylazine.
3. Inject the mouse intraperitoneally with the calculated mixture of ketamine and xylazine and place it back in the cage.
4. Wait 5–10 min until the anesthesia has set in, then place the mouse with its back on the cage grid and soak the abdomen with 70% ethanol.
5. Place the mouse on its back on the heating plate and fix it to the plate using fixation plasters over the extremities.
6. Test the flexor reflex and confirm absence of any movements, then open the abdominal cavity of the mouse.
7. Push the intestine to one side using a cotton swab to expose the liver and vessels.
8. Place a thread of suture material below the portal vein using two curved pointed forceps and make a loose knot. Do not tie the knot but leave the suture material as a loop.
9. Make another loop under the caudal vena cava below the liver and above the renal vein using surgical suture material.
10. Insert a 26 G venous catheter into the portal vein with the help of the stereo microscope (Figure 1, position 4).
11. Fix the indwelling venous catheter by tightening the knot of suture material. Wait until the catheter fills with blood and a drop forms in the front of the Luer-Lock connection.
12. Switch off the pump and connect the pump tubing to the venous catheter.
13. Switch on the pump and cut open the caudal vena cava below the renal vein using a dissecting scissor to exsanguinate the liver. Place a paper towel next to the mouse to soak up the blood and solution (Figure 1, position 5). At this point, the anesthetized mouse dies from blood loss.
14. Flush the liver with solution 1 until it is free of blood. Meanwhile, open the thorax of the mouse and place a suture under the caudal vena cava and make a loose knot.
15. Insert another indwelling venous catheter into the caudal vena cava via the right atrium of the heart and secure it with the tightened loop of suture material (Figure 1, position 6).
16. Stop the pump once the liver is free of blood and then insert the pump tubing from solution 1 into solution 2. Tighten the knot on the caudal vena cava under the liver.
17. Switch on the pump and flush the liver with solution 2. Connect a pump tubing to the second indwelling venous catheter when a drop forms in the front of the Luer-Lock connection. Fix the pump tubing so that it protrudes over the table and points down at the edge of the table. Monitor and note the system pressure in cm water column. Collect the flow-through for 1 min to define minute 0 (Figure 1, position 7).
18. After rinsing the liver, switch off the pump and transfer the pump tubing from solution 2 into solution 3 to start the substrate perfusion.
19. Switch on the pump and collect the flow-through into a new reaction tube every minute for 30 min. Monitor and note the pressure of the system at the end of the 30 min.
20. After 30 min, switch off the pump and collect the remaining perfusate still in the pump tubing by removing the indwelling venous catheter in the heart (minute 31).

### 3.3 Preparation of the samples for LC-MS/MS measurement (Step III)

1. Add 400 µL of the post-perfusion processing solution (solution 4) to 200 µL perfusate.
2. Vortex the mixtures and centrifuge at 16,000 × *g* for 15 min.
3. Evaporate 350 µL from each supernatant to dryness under nitrogen flow at 40 °C.
4. Dissolve the pellet in 200 µL 0.1% formic acid and centrifuge it at 16,000 × *g* for 10 min.
5. Transfer 100 µL of the supernatant into an LC-MS/MS vial with insert and inject it into the LC-MS/MS system as described below.

### 3.4 Optimizations

I.1 The water bath was preheated to 42 °C so that the solution would reach the liver at the physiological temperature of 37 °C. This eliminates the need for additional heating of the entire system.
I.3 The flow rate was set to 2 mL/min based on the flow rate in the mouse portal vein (1.6–2.3 mL/min; Xie et al., 2014).
I.4 The T-shaped glass tubing was used to monitor the pressure in the liver. This setup allows continuous monitoring of the system’s pressure during the experiment. Without monitoring, an increase in pressure would only become apparent through liver swelling, which could ultimately lead to liver rupture.
I.9 The heating plate was used to maintain the physiological body temperature during the experiment.
I.10 Measuring the drug concentration in solution 3 allows verifying the solution in terms of correct concentration and correct drug of interest.
II.4 Without the ethanol moistening, the mouse’s fur would stick everywhere and impair the view of the veins.
II.7 The intestine is not cut out because it would lead to premature bleeding.
II.8 The loose knot of suture material is used to secure the indwelling venous catheter so that it does not slip out of the vein.
II.9 The suture material around the caudal vena cava between liver and renal vein is tied, as it prevents the backflow into the kidneys.
II.10 A 26 G venous catheter was in our hands the most suitable size for the small mouse veins.
II.11 Waiting until the blood forms a drop out of the Luer-Lock connection reduces air bubble formation in the beginning of the perfusion.
II.13 The caudal vena cava was cut open to ensure a faster exsanguination of the liver.
II.14 The flush out should take 6–8 min, but can be prolonged when the preparation takes longer.
II.15 The venous catheter is placed into the caudal vena cava via the right atrium, as the heart additionally secures the venous catheter.
II.17 The tube was fixed so that it protrudes downwards over the edge of the table, as this makes it easier to collect the flow-through.
II.19 The perfusion time was set to 30 min, as at 40 min the liver began to suffer damage (hypoxic; white spots).

### 3.5 Troubleshooting

Advice for troubleshooting can be found in Table 4.

**TABLE 4:** Troubleshooting.

Step	Problem	Possible reason	Solution
II.6	Mouse was not anesthetized properly	Mouse had too much stress before	Inject 10% of the dose of ketamine again; change ketamine and xylazine to a new aliquot; try to minimize stress for the mouse
II.8 and II.9	The vessel is punctured when putting a thread under it	The surgical suture material or the forceps were placed too close to the vessels	Stop bleeding by pressing a cotton swab on it and quickly place the venous catheter in the portal vein and start perfusion
II.10	The vessel is punctured while placing the venous catheter	The vessel moved with the needle and it was then pressed too tightly	Use a cotton swab to secure the vessel and then place the venous catheter
II.11	Blood is getting into the Luer-Lock connection too slowly	The mouse is too young or small and has a lower blood volume. The venous catheter is placed too close to the vessel	Slightly loosen the knot and move the indwelling venous catheter. Take a pipette and fill the Luer-Lock connection with solution 1
II.14	The liver has a spot where it is not bled out	There was a small thrombus in the animal	Press down the opened caudal vena cava with a cotton swab and apply some pressure to the liver
II.14 and II.15	Pressure increases constantly	The indwelling venous catheter is attached to the vessel or is close to the liver The catheter between heart and liver has slipped out	Loosen the knot slightly and move the indwelling venous catheter slightly so that it is back in the correct position
II.16	Air bubbles in the system	The gasified medium sometimes has bubbles in it. Sometimes there are bubbles when the solutions are switched	Small and medium bubbles are directed through the T-piece into the tube, where the pressure is measured, which then does not interfere If there are bubbles in the tubing to the liver, the tube can be temporarily detached from the cannula and the bubble removed (rare)
II.18	System is leaking or collection volume is smaller than 2 mL	The indwelling venous catheter between heart and liver has punctured the caudal vena cava	Try to find the leak and close it with a second thread; if this is not possible, a small leak (1.8 mL instead of 2 mL) is acceptable

### 3.6 Step times for one mouse

Step I: preparing the perfusion system: 1 h.

Step II: preparation of the mouse and perfusion: 2 h.

After step II, the samples can be stored at −20 °C for a short time, or at −80 °C for a longer time.

Step III: perfusate sample preparation 4 h.

### 3.7 Quantification of lucifer yellow

The concentrations of the fluorescence dye, lucifer yellow, in liver perfusates were measured using a Tecan Ultra Microplate Reader (Tecan Group AG, Männedorf, Switzerland). For each time point, 200 µL of perfusate was loaded in duplicates into a Nunc™ Flat Bottom Black MicroWell™ plate (Thermo Fisher Scientific, Waltham, United States; Cat. Nr. 237105). Measurement was conducted via top reading with an excitation wavelength set at 427 nm and emission wavelength at 540 nm. The instrument gain was manually set to 100, and each well was read four times to obtain fluorescence intensity values. Data acquisition and analysis were performed using the Tecan software i-Control^TM^ 1.6.

### 3.8 Quantification of substrate concentrations by LC-MS/MS

Substrate concentrations in liver perfusates were measured using LC-MS/MS. To this end, 10 µL for proguanil and cycloguanil and 15 µL for morphine and codeine samples were injected into the LC-MS/MS system. LC-MS/MS analysis was performed using an LC-30AD binary pump, an SIL-30AC autosampler, and a CTO-20AC column oven (Shimadzu Corporation, Kyoto, Japan) connected to a Sciex QTRAP 4000 triple quadrupole mass spectrometer (AB SCIEX, Darmstadt, Germany). Electrospray ionization (ESI) was used for MS detection in positive ion mode.

For codeine and morphine, the source parameters were set as follows: source temperature, 500 °C; ion spray voltage, 5.5 kV; collision gas, medium; curtain gas, 32 psi; ion source gas 1 and 2, 50 psi; dwell time, 150 msec; entrance potential (EP), 10 V; collision cell exit potential (CXP), 10 V. For proguanil, the source parameters were set as follows: source temperature, 450 °C; ion spray voltage, 5.5 kV; collision gas, medium; curtain gas, 35 psi; ion source gas 1, 55 psi and ion source gas 2, 60 psi; dwell time, 100 msec; entrance potential (EP), 10 V; collision cell exit potential (CXP), 10 V. Declustering potential (DP) and collision energy (CE) were optimized for each substrate individually (Table 3).

Chromatographic separation of the substances was achieved using a Brownlee SPP RP-Amide column (4.6 mm × 100 mm, 2.7 μm; PerkinElmer, Rodgau Germany). Solvent A consisted of 77% acetonitrile, 13% methanol and 10% 0.1% formic acid in water (all v/v). Solvent B consisted of 0.1% formic acid in water. For codeine and morphine, separation was achieved at a flow rate of 0.35 mL × min^−1^ using an isocratic method for 11.5 min with a mobile phase containing 3.3% solvent A. For proguanil, separation was achieved at a flow rate of 0.5 mL × min^−1^ using an isocratic method for 5 min with 30% solvent A. The column temperature was set to 40 °C and the autosampler temperature was 5 °C. Analyst 1.7.1 software (AB SCIEX) was used for data analysis.

### 3.9 Data analysis

For visualization of the substrate concentrations in the perfusate over the entire perfusion and for calculation of the resulting area under the curve (AUC), GraphPad Prism version 8.0.2 (GraphPad Software, Inc.) was used. The AUC_0-30min_ from the wild-type (FVB) mice and the Oct1/2 knock out (Oct1/2 KO) mice was compared using the Mann-Whitney U test in SPSS version 29 (IBM SPSS Statistics).

## 4 Results

### 4.1 Initial parameter optimization of the perfusion model using a fluorescent dye

As a first step, we tested the functionality of the system and optimized parameters such as flow rate and sampling intervals based on the time to reach maximum concentration and the subsequent return to baseline after substrate washout. To this end, we used 100 nM lucifer yellow as a fluorescent tracer, which is not expected to undergo metabolism or to be subject to active or passive transport processes in the mouse liver.

We perfused the liver with a flow rate of 2.5 mL/min and collected the perfusate every 2.5 min for 37.5 min. With a constant substrate perfusion for 20 min, it took around 7.5 min to reach the maximum concentration and another 7.5 min to nearly reach baseline levels after substrate was washed out with HBSS (Supplementary Figure S2). Based on these pilot experiment (n = 1), we optimized the following parameters: the flow rate was changed to 2 mL/min to better reflect the normal portal vein flow rate (1.6–2.3 mL/min) (Xie et al., 2014). Also, the time interval for sample collection was reduced to 1 min, to enable more precise detection of small differences. The total perfusion time was shortened to 30 min since the liver showed hypoxic damage (white spots, data not shown) after 40 min.

### 4.2 Using the method to analyze the effect of Oct1 knockout on the first-pass metabolism of codeine

**FIGURE 3:**
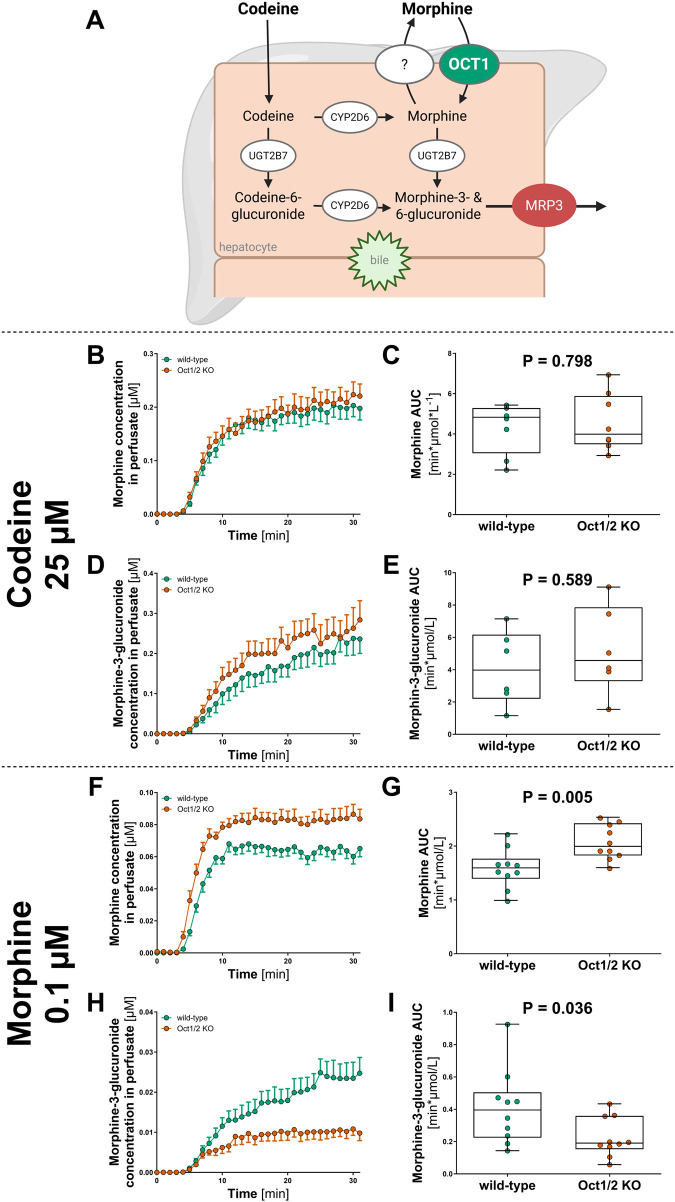
Effect of Oct1 knockout on the first-pass metabolism of codeine and morphine Schematic overview of the hepatic metabolism of codeine and morphine, adapted from Tzvetkov et al. (Tzvetkov et al., 2013) OCT1 is expected to mediate hepatocellular uptake of morphine but not of codeine, as the higher lipophilicity of codeine allows passive diffusion into hepatocytes. **(A–D)** Concentrations of morphine **(B,C)** and its major metabolite morphine-3-glucuronide **(D,E)** was measured after liver perfusion of Oct1/2 knockout (red) and wild-type (green) mice with 25 µM codeine. Panels B and D show the time course of morphine and morphine-3-glucuronide concentrations measured in the perfusate, while panels C and E present the descriptive statistics of the experiments. Shown are median ± quantiles of the AUC_0-30min_ (n = 8). **(F–I)** The concentration of morphine **(F,G)** and its metabolite morphine-3-glucuronide **(H,I)** was measured after director perfusion of 0.1 µM morphine. Panels F and H show the time course of morphine and morphine-3-glucuronide concentrations measured in the perfusate, while panels G and I present the descriptive statistics of the experiments. Shown are median ± quantiles of the AUC_0-30min_ (n = 10). The statistical analyses were performed using Mann-Whitney U test with significance threshold of 0.05.

We applied the perfusion method to analyze the effect of Oct1 knockout on the first-pass metabolism of several drugs that are well known substrates of OCT1: morphine (alone or as an active metabolite of codeine), proguanil and cycloguanil (alone and as an active metabolite of proguanil). Codeine is metabolized to morphine in the hepatocytes (Figure 3A). Both codeine and morphine are metabolized to their 3- and 6-glucuronides by UGT2B7. In mouse, only the morphine-3-glucuronide is produced (Kuo et al., 1991; Zelcer et al., 2005; Gabel et al., 2022). After formation, morphine partially exits the hepatocytes into the blood stream (via a yet unknown transporter) and can be taken back up by OCT1 (Tzvetkov et al., 2013). We applied the established perfusion model to confirm the role of Oct1 in this process. To this end, we compared the perfusion of codeine and morphine between Oct1/2 knockout and wild-type mice.

We did not observe significant effects of Oct1 knockout when perfusing of 25 µM codeine (n = 8 each genotype, Figures 3B–E). After codeine perfusion, morphine, morphine-3-glucuronide, and codeine-6-glucuronide were detected. However, neither the formation of morphine (Figures 3B,C) nor that of its metabolite morphine-3-glucuronide (Figures 3D,E) differed between Oct1 wild-type and knockout. Similarly, no OCT1-dependent differences were observed in the production of the direct codeine metabolite codeine-6-glucuronide (Supplementary Figure S3).

However, when we perfused 0.1 µM morphine directly (n = 10 each genotype), we observed significant genotype-dependent differences for both morphine and its metabolite morphine-3-glucuronide (Figures 3F–I). Perfusates from Oct1-knockout animals had higher steady state concentrations of morphine (30% higher AUC_0-30min_, P = 0.002; Figures 3F,G) and lower concentrations of morphine-3-glucuronide (45% lower AUC_0-30min_, P = 0.03; Figures 3H,I).

### 4.3 Using the method to analyze the effect of Oct1 knockout on the first-pass metabolism of proguanil

**FIGURE 4:**
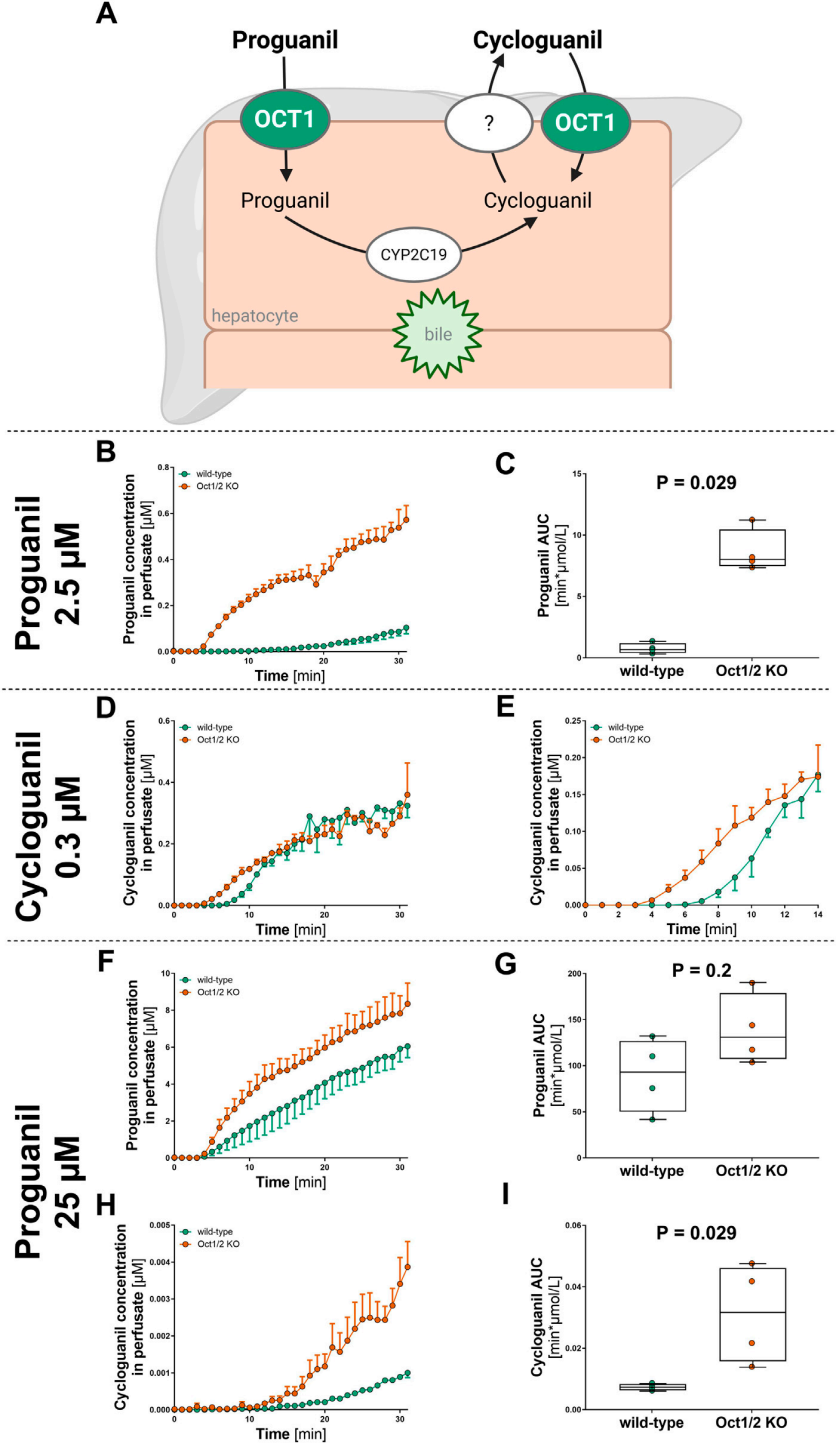
Effect of Oct1 knockout on the first-pass metabolism of proguanil and cycloguanil Schematic overview of the hepatic metabolism of proguanil and cycloguanil **(A)**, adapted from Matthaei et al. (Matthaei et al., 2019). OCT1 is expected to mediate hepatocellular uptake of proguanil and cycloguanil. **(B,C)** Concentration of proguanil was measured after liver perfusion of Oct1/2 knockout (red) and wild-type (green) mice with 2.5 µM proguanil. Panel B show the time course of proguanil concentration measured in the perfusate, while C present the descriptive statistics of the experiments. Shown are median ± quantiles of the AUC_0-30min_ (n = 4). **(D,E)** Concentration of cycloguanil was measured after liver perfusion of Oct1/2 knockout (red) and wild-type (green) mice with 0.3 µM cycloguanil. Panel D show the time course of cycloguanil concentration measured in the perfusate over 30 min, while E shows an enlarged view of the first 14 min (n = 3). **(F–I)** Concentration of proguanil **(F,G)** and cycloguanil **(H,I)** was measured after perfusion of 25 µM proguanil. Panel F and H show the time course of proguanil and cycloguanil concentration in the perfusate, while panel G and I present the descriptive statistics of the experiments. Shown are median ± quantiles of the AUC_0-30min_ (n = 4). The statistical analyses were performed using Mann-Whitney U test with significance threshold of 0.05.

In a similar experiment, we applied the perfusion model to analyze the effect of Oct1 knockout on the first-pass metabolism of proguanil. Proguanil is metabolized to its active metabolite cycloguanil by CYP2C19 in hepatocytes (Figure 4A Hoskins et al., 1998). Both proguanil and cycloguanil are substrates of OCT1 (Matthaei et al., 2019), which mediates their uptake into the hepatocytes. After formation, cycloguanil may exit the hepatocytes via a yet unknown transporter and may be taken back up by OCT1. We tested this hypothesis using the perfusion model by comparing the perfusion of the parent drug proguanil with the direct perfusion of its metabolite cycloguanil in Oct1/2 knockout and wild-type mice.

When proguanil was perfused at concentrations comparable to those observed in human plasma (2.5 µM), we observed a pronounced effect of Oct1/2 knockout on proguanil concentrations. The proguanil concentration in the perfusate exiting the livers of Oct1/2 knockout mice was approximately 1,000% higher than that in wild-type mice (AUC_0-30min_, p = 0.03; Figures 4B,C; n = 4). However, at this concentration, the formation of the active metabolite cycloguanil could not be detected. When cycloguanil itself was directly perfused (Figure 4D; n = 3), there was a trend toward a faster increase in perfusate concentrations during the first 13 min in Oct1/2 knockout mice (Figure 4E), although no significant differences in AUC or steady-state concentrations were observed.

To enable cycloguanil detection after proguanil perfusion, the proguanil concentration was increased to 25 µM. Proguanil levels in the perfusate remained higher in Oct1/2 knockout mice than in wild-type mice, although the difference was no longer significant (340% higher AUC_0-30_min, Figures 4F,G; n = 4). More importantly, under these conditions, cycloguanil formation became detectable, and the concentrations of the produced and excreted in the perfusate cycloguanil were significantly higher in Oct1/2 knockout mice (4.2-fold higher AUC_0-30min_, Figures 4H,I; n = 4).

These results demonstrate that our perfusion system is able to simulate the first-pass metabolism in an *ex vivo* setting and help gain additional information about the role of drug transporters in pharmacokinetics.

### 4.4 Reproducibility of the perfusion conditions

To assess the variability of the method, we focused on the pressure within the system as a well-measurable and informative parameter for stable and complete liver perfusion. The pressure increased by a maximum of 6% from the start to the end of perfusion at minute 30 (Table 5), indicating stable perfusion over the tested period. The method also proved to be reproducible, with the coefficient of variation for both the initial and final pressure ranging between 7% and 19%.

**TABLE 5:** Pressure differences at the beginning and end of the perfusion [mbar]. Shown are means ± SEM.

Study	Strain	n	Pressure				Coefficient of variation [%]	
			Beginning	End	Delta	Delta [%]	Beginning	End
Codeine and morphine	wild-type	22	17.22 ± 0.66	18.09 ± 0.74	0.87 ± 0.26	5.02 ± 1.54	17.9	19.1
	Oct1/2 KO	22	17.29 ± 0.45	18.32 ± 0.60	1.02 ± 0.31	5.82 ± 1.81	12.2	15.4
Proguanil and cycloguanil	wild-type	9	18.46 ± 0.42	18.90 ± 0.44	0.44 ± 0.11	2.40 ± 1.04	6.9	7.0
	Oct1/2 KO	9	18.63 ± 0.54	18.74 ± 0.53	0.11 ± 0.16	0.63 ± 0.90	8.6	8.5

KO, knockout.

The increase in pressure was less pronounced, and the variability was lower in the second study (proguanil/cycloguanil) compared to the first study (codeine/morphine), suggesting a potential for further improvement based on the researcher’s learning curve.

To minimize inter-day variability, the experiment was designed to use a knockout and a wild-type mouse on the same day. Indeed, the pressure variability, measured as the coefficient of variation, was on average twice as high between days compared to within a single day (Table 6). Similarly, the variability in the first study (codeine/morphine) was higher than in the second study (proguanil/cycloguanil), both between and within days.

**TABLE 6:** Pressure differences at the beginning and end of the perfusion as coefficient of variation for intra-day and inter-day comparison. Shown are means ± SEM.

Study	n (days)*	Intra-day coefficient of variation [%]		Inter-day coefficient of variation [%]	
		Beginning	End	Beginning	End
Codeine and morphine	22	6.69 ± 1.24	7.85 ± 1.01	15.1	17.1
Proguanil and cycloguanil	9	3.15 ± 0.83	2.70 ± 0.69	7.6	7.5

* two mice per day: one wild-type and one Oct1/2 KO.

## 5 Discussion

In this study, we present an *ex vivo* perfusion model that allows the simulation of first-pass hepatic effects on systemic drug concentrations. The method proved successful in simulating systemic concentrations of both the parent drug and its major metabolites. Specifically, we used the model to assess the role of the hepatic uptake transporter Oct1 in the metabolism and systemic concentrations of codeine, morphine, and proguanil.

Adapting the well-established rat liver perfusion model to mice represents a significant advancement, as it allows the use of knockout animals to study the effects of individual genes. Despite the progress in CRISPR-Cas technology, the number of available knockout mouse models still far exceeds that of knockout rat models. In this work, we adapted a well-established rat liver perfusion protocol of Seglen (1976), originally used for isolating primary hepatocytes, to create a mouse model suitable for direct liver analyses.

Following key modifications of the Seglen protocol were critical for our application: reversing the direction of perfusion, adjusting the perfusion flow rate, and the preheating of perfusion solutions. We adjusted the perfusion direction to start in the portal vein and end in the heart, following the physiological flow pattern, instead of the previously described approach of starting in the caudal vena cava and ending in the portal vein (Aiken et al., 1990). Accordingly, perfusates were collected via the caudal vena cava between the heart and liver, rather than between the kidney and liver as outlined in the original protocol. This was adopted from the protocol used by Choi et al. (2019). The perfusion flow rate was set to 2 mL/min to closely resemble the physiological blood flow rate in the mouse portal vein. Additionally, the perfusion solutions were preheated to 42 °C, and the mouse was placed on a heating plate maintained at 37 °C. This approach simplified the procedure by eliminating the need for precise temperature control throughout the entire system. Preheating the solutions to 42 °C ensured that they reached a physiological temperature of approximately 37 °C upon entering the mouse liver. This temperature was verified during method optimization using a roasting thermometer.

The scientific question addressed to illustrate the use of the liver perfusion model was the role of the hepatic uptake transporter Oct1 in the metabolism of weak bases that can partially diffuse into hepatocytes and are further converted to metabolites excreted into the circulation. When the parent drug is an Oct1 substrate, such as morphine, loss of Oct1 activity is expected to reduce metabolite formation. Indeed, after direct morphine perfusion, morphine 3-glucuronide production was decreased (Figures 3H,I), consistent with previous findings in humans (Venkatasubramanian et al., 2014).

In contrast, no significant effect was observed after codeine perfusion, where morphine-3-glucuronide levels showed even a minor, nonsignificant increase. There is no evidence that OCT1 functions as an efflux transporter for morphine, either *in vitro* or in OCT1-deficient humans (Tzvetkov et al., 2013; Venkatasubramanian et al., 2014; Tzvetkov, 2017). A likely explanation is that codeine was administered at relatively high concentrations, leading to high morphine production under these conditions. Because morphine exhibits moderate passive permeability, OCT1 contributes substantially to uptake only at lower concentrations (Tzvetkov et al., 2013, and in our study - Figure 3G). Thus, the lack of effect after codeine administration likely reflects high morphine exposure rather than the use of the prodrug itself. Alternative, compensatory mechanisms in Oct1-deficient mice, such as increased expression of efflux transporters (e.g., Mrp3), may also contribute, although this remains unconfirmed.

Regarding the effects of Oct1 deficiency on proguanil liver passage and cycloguanil formation, the impact of hepatic uptake was weaker than expected when cycloguanil was directly perfused (Figures 4D,E). However, when cycloguanil was generated intracellularly from proguanil (25 µM perfusion; Figures 4H,I), cycloguanil concentrations in the perfusate were markedly and significantly higher in Oct1/2 knockout mice than in wild-type controls.

Two conclusions can be drawn from these findings. First, in the absence of OCT1, the reduced reuptake of newly formed cycloguanil into hepatocytes appears to outweigh the reduced uptake of its precursor proguanil via Oct1, leading to a net increase in cycloguanil concentration in the perfusate of the Oct1 knockout mice. This contrasts with human data showing lower systemic cycloguanil levels in individuals carrying OCT1 alleles with reduced activity (Matthaei et al., 2019). Species-specific differences in transport kinetics or in the spatial expression of OCT1 between mouse and human liver may explain this discrepancy, but experimental data are needed to confirm it. Second, the impact of Oct1-mediated uptake may be greater for metabolites formed and released by hepatocytes than for compounds introduced externally via perfusion. This could be due to steeper concentration gradients driving OCT1 transport when cycloguanil is locally produced and released near the transporter in the space of Disse, rather than delivered from the sinusoidal blood.

The liver perfusion model could be further developed. In this study, we demonstrated liver perfusion of parent drug-to-metabolite pairs like codeine and morphine, and proguanil and cycloguanil. This method can also be applied to other parent drug-to-metabolite pairs, such as debrisoquine/hydroxy-debrisoquine and tramadol/O-desmethyltramadol (Saadatmand et al., 2012; Stamer et al., 2016). It enables us to differentiate the role of Oct1 in the uptake of the parent drug compared to its metabolite or may help us to identify other transporters involved, e.g., by using selective inhibition in the Oct1 knockout model. The approach can be extended by using mice with other transporter knockouts (e.g., MATE1, OATP1B1, or efflux transporters like MRP3) to study their impact on metabolism.

A time-resolved collection of bile would significantly improve the method. This is generally possible (Bertolini et al., 2022). However, this procedure is highly delicate and requires a catheter smaller than 26 G, as described by Chen et al. (2023). This issue is less problematic in rats, as they lack a gallbladder and instead have enlarged bile ducts, which facilitate catheterization (Newell et al., 1990; Tønsberg et al., 2010; Claussen et al., 2020).

Another optimization may be in reducing the number of mice per experiment. We previously established a cocktail of OCT1 substrates (Rönnpagel et al., 2025) that enables analyzing up to ten different substrates in one experiment. The cocktail is a versatile tool to analyze different aspects of drug metabolism and transport and may be directly applied using the already developed LC-MS/MS analytic procedure (Rönnpagel et al., 2025) both in Oct1 knockout animals and for drug-drug interactions. We already successfully used the method for analyzing the effects of Oct1 on hepatic first-pass of berberine (Blöcher et al., 2024).

Theoretically, this method could be combined with *ex vivo* models to assess the intestinal contribution to first-pass metabolism, such as the Ussing chamber. However, using mouse intestine in a Ussing chamber is also challenging and may require optimization.

The mouse liver perfusion model presented here provides a valuable complement to existing 2D and 3D systems for studying hepatic drug transport and metabolism. Traditional 2D cultures lack physiological flow and cell–cell interactions, resulting in altered transporter expression and absent bile secretion. Despite these limitations, they remain accessible and suitable for basic uptake and metabolism studies (Wilkening et al., 2003; Olsavsky et al., 2007; Gerets et al., 2012; Jouan et al., 2016; Malinen et al., 2019). Three-dimensional cultures better reproduce hepatic microarchitecture and can integrate microfluidic flow, although bile excretion is still restricted and technical demands are higher. Organ-on-chip systems further enhance physiological relevance by mimicking blood flow and concentration gradients, yet transporter and enzyme expression profiles remain suboptimal, and hepatic zonation and bile secretion are not fully recapitulated (Ehrlich et al., 2019). Precision-cut liver slices (PCLS) maintain native architecture and cellular diversity for metabolism and toxicity testing, but they lack vascular perfusion and bile collection. Their efficiency allows multiple analyses from one liver, reducing animal use. Ultimately, no single model fully reproduces hepatic physiology; model selection should depend on the experimental objective (Dewyse et al., 2021). The limited availability of human hepatocytes and non-parenchymal cells continues to be a constraint, underscoring the need for alternative systems such as humanized mice to bridge the gap between *in vitro* and *in vivo* liver research. Liver perfusion, in contrast, preserves the full hepatic microarchitecture, including all liver cell types, vascular perfusion, and cellular integrity. However, it requires considerable technical expertise and a higher number of animals compared to other methods. Moreover, the translational relevance of mouse data to humans must be carefully considered.

The data obtained using the mouse model described here should be carefully interpreted regarding their translatability to humans. Species differences between rodents and humans, particularly in single aspects of organ physiology (Floerl et al., 2020; Hagenbuch et al., 2025; Martignoni et al., 2006) and the sex-specific expression of key drug-metabolizing enzymes and transporters, are well documented (Breljak et al., 2013; Gerges and El-Kadi, 2023). Notably, Oct1, the transporter analyzed in this study, differs between species in both organ expression and substrate selectivity. In humans, Oct1 is expressed only in the liver (Gorboulev et al., 1997; Zhang et al., 1997), whereas in rodents and dogs, it is present in both the liver and kidney (Green et al., 1999; Schmitt et al., 2003; Meyer et al., 2022a). Additionally, the uptake kinetics of OCT1 vary between mice and humans in a substrate-specific manner (Dresser et al., 2000; Meyer et al., 2020; 2022b).

**FIGURE 5:**
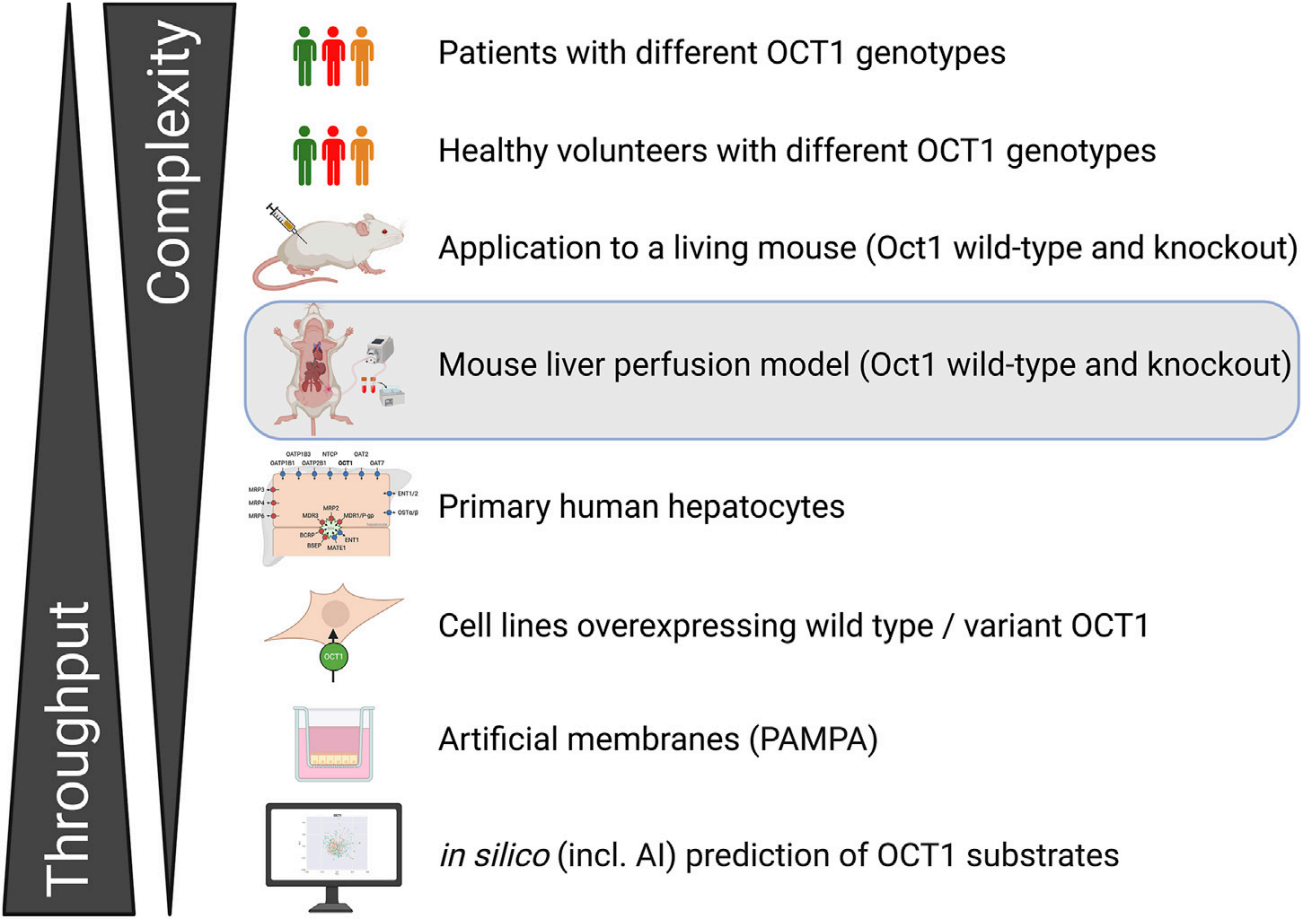
Example of a potential integration of the liver perfusion model into the pipeline established to identify and demonstrate the clinical relevance of new OCT1 substrates.

This model should not be considered as a stand-alone approach, but rather as an addition to a more comprehensive pipeline for analyzing the role of hepatic transporters or metabolizing enzymes. Such a pipeline should also incorporate human preclinical and clinical data. An example from our laboratory is provided in Figure 5.

Additional limitations that require further optimization include the measurement of intrahepatic concentrations of the drug and its metabolite. While the method performed well for perfusate analysis, it was less reliable for intrahepatic concentrations. The measured intrahepatic concentrations varied greatly, even between different regions of the same liver.

In conclusion, we successfully developed an *ex vivo* liver perfusion model to simulate hepatic first-pass effects on systemic drug concentrations. The method proved effective for analyzing the role of Oct1 in the hepatic uptake prior to metabolism of codeine, morphine, and proguanil. Despite its advantages, careful interpretation is necessary due to species differences between mice and humans, particularly regarding OCT1 expression and function. Future improvements should focus on enhancing intrahepatic concentration measurements and integrating bile collection to increase data accuracy and translatability.

## Data Availability

The raw data supporting the conclusions of this article will be made available by the authors, without undue reservation.
