# Supplementary material for: Ex vivo perfusion model of mouse liver and its application to analyze the effects of OCT1 deficiency
Source: Front Pharmacol. 2025 Nov 14;16:1629421. doi: 10.3389/fphar.2025.1629421 (PMC12660287; doi:10.3389/fphar.2025.1629421)
Supplement: Supplementary file 1 [file DataSheet1.pdf]

Supplementary Material

1 Supplementary Figures

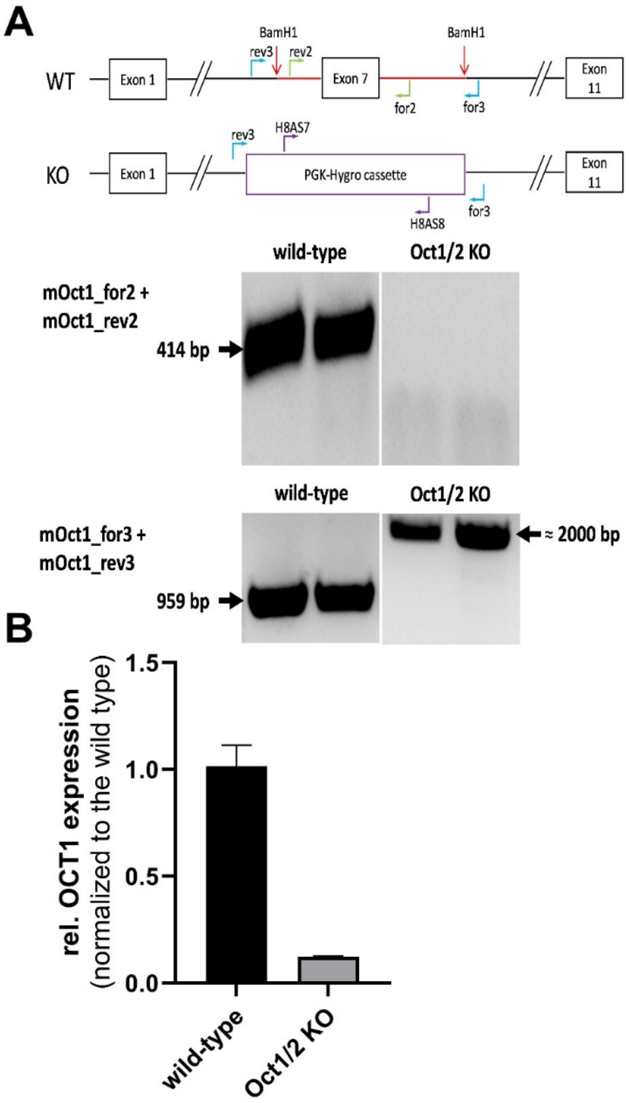

Supplementary figure S1. Validation of the Oct1 knockout at DNA (A) and RNA (B) level. The DNA and RNA were isolated from livers of the wild type and OCT1/2 knock out (OCT1/2 KO) mice. The DNA was tested with primer designed to bind in the BamH1 restriction site used by Jonker et al. to knock out Oct1 (for2+rev2). The second designed primer binds before the BamH1 restriction site and show a smaller fragment for the wild type (959 bp) and a bigger fragment for the knockout (around 2000 bp). The RNA was tested in a Real Time PCR using the mSlc22a1 (Mm00456303_m1) assay (n=4).

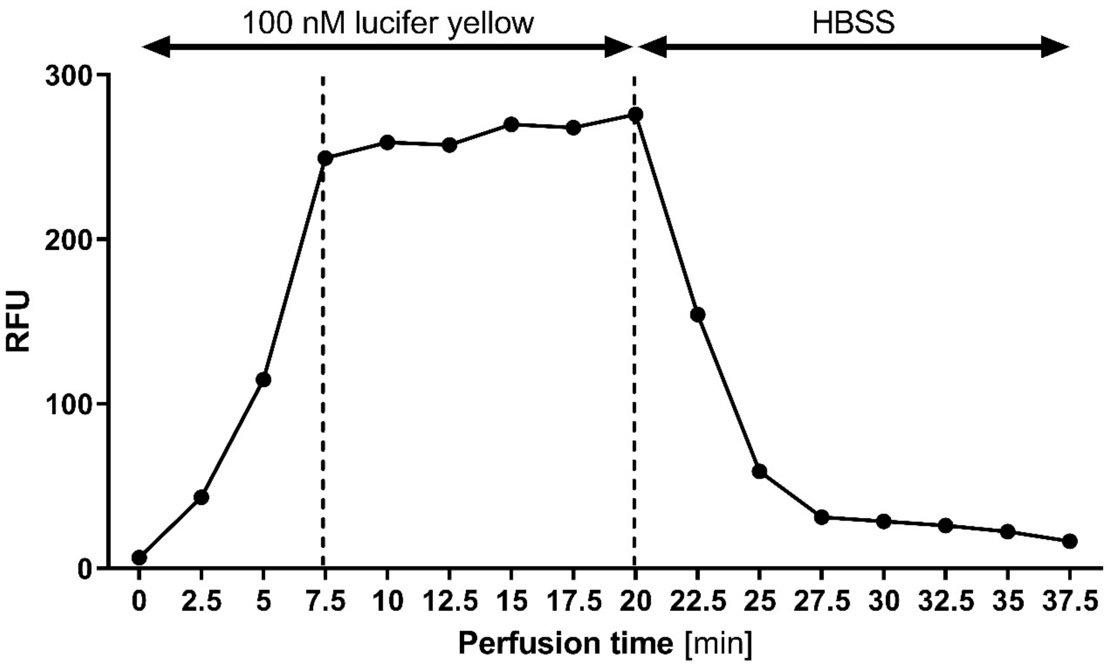

Supplementary figure S2. Initial characterization and optimization of the mouse liver perfusion model with 100 nM lucifer yellow over 37.5 minutes. The flow rate was set to 2.5 mL/min and the perfusate was collected every 2.5 minutes. After 20 minutes, perfusion of the substrate was stopped and changed to perfusion with buffer (HBSS) only. n = 1

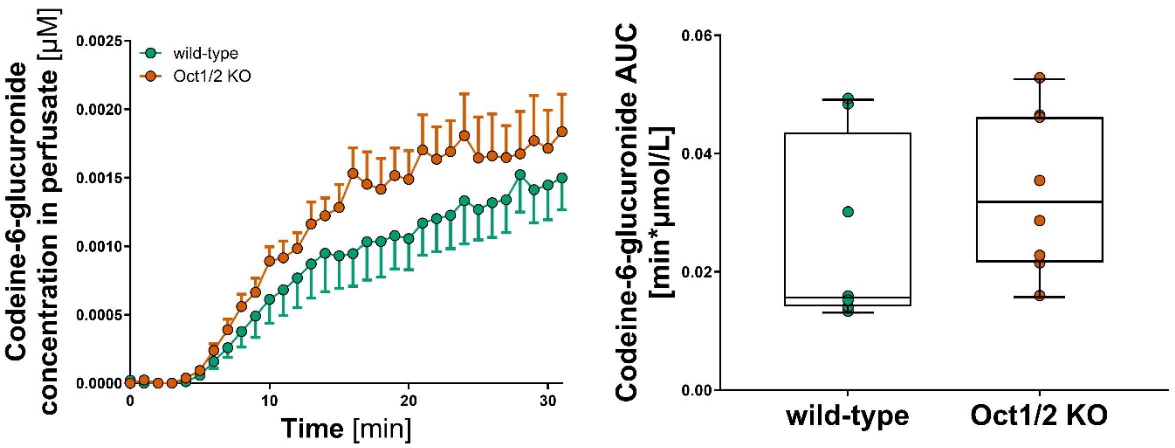

Supplementary figure S3. Effect of Oct1 knockout on the first-pass metabolism of codeine. The concentration of codeine-6-glucuronide was measured after liver perfusion of Oct1/2 knockout (red) and wild-type (green) mice with 25 µM codeine (n = 8) over 30 minutes (left side). The $AUC_{0\text{-}30min}$ was calculated for codeine-6-glucuronide (right side). Shown are median ± quantiles.
